# Enhanced efficacy of photodynamic therapy by inhibiting ABCG2 in colon cancers

**DOI:** 10.1186/s12885-015-1514-4

**Published:** 2015-07-07

**Authors:** Ju Hee Kim, Jae Myung Park, Yoon Jin Roh, In-Wook Kim, Tayyaba Hasan, Myung-Gyu Choi

**Affiliations:** 1 Catholic Research Institute of Medical Science, The Catholic University of Korea, Seoul, South Korea; 2 Wellman Center for Photomedicine, Department of Dermatology, Massachusetts General Hospital, Harvard Medical School, Boston, MA USA; 3 Division of Gastroenterology, Department of Internal Medicine, Seoul St. Mary’s Hospital, The Catholic University of Korea, 222 Banpo-daero, Seocho-gu, Seoul, 137-071 South Korea

**Keywords:** Photodynamic therapy, Colon cancer, Protoporphyrin, ATP-binding cassette transporters, Photosensitizing agents

## Abstract

**Background:**

Photodynamic therapy (PDT) contains a photosensitizing process, which includes cellular uptake of photosensitizer and delivery of light to the target. ATP-binding cassette subfamily G2 (ABCG2) regulates endogenous protoporphyrin levels. In human colon cancers, it is not fully examined the role of ABCG2 in porphyrin-based photodynamic therapy.

**Methods:**

SW480 and HT29 cells were selected because they showed low and high ABCG2 expression levels, respectively. Pyropheophorbid-a (PPa) was used as a photosensitizer. Cells were exposed to a 670 nm diod laser. Cell viability and necrosi apoptosis was examined. Production level of singlet oxygen was detected with the photomultiplier-tube s/ -based singlet oxygen detection system.

**Results:**

SW480 cells, which expressed lower level of ABCG2, showed the higher uptake of PPa than HT-29 cells. The uptake level of PPa was significantly correlated with the decreased cell viability after PDT. Pretreatment with a ABCG2 inhibitor, Ko-143, significantly enhanced the PDT efficacy in HT29 cells compared to vehicle-pretreated cells. To confirm the ABCG2 effect on PDT, we established ABCG2 over-expressing stable cells in SW480 cells (SW480/ABCG2). Furthermore, SW480/ABCG2 cells showed significantly decreased PDT effect compared to the control cells. The increased or decreased cell survival was significantly correlated with the production level of singlet oxygen after PDT.

**Conclusion:**

ABCG2 plays an important role in determining the PDT efficacy by controlling the photosensitizer efflux rate. This implies the control of ABCG2 expression may be a potential solution to enhance photosensitivity.

## Background

Photodynamic therapy (PDT) is a modality that uses a photosensitizer that is selective to target cells, followed by exposure to a given specific light source [1, 2]. Photosensitizer is excited by irradiation, producing reactive oxygen species such as singlet oxygen, other free radicals and cause necrosis or apoptosis of the target cells [3]. PDT is indicated in the treatment of various cancers and is known to be particularly effective in basal cancer cells such as skin cancer [4].

Pheophorbide a (PPa) is a chlorophyll-based photosensitizer which is localized to the mitochondria [5]. Many studies reported that PPa showed an anti-tumor effect in vivo on human lung, liver, and skin cancer [6–8]. Irradiated PPa produced singlet oxygen which rapidly induced depolarization of mitochondrial membrane potential and tumor cell death. PDT using PPa has been attempted on hepatocarcinoma, Jurkat leukemia cell, pigmented melanoma, and colon cancer cells [9–11]. PPa had structural similarities with porphyrin which was the most commonly used photosensitizer [12].

The main problem in the chemotherapy of cancer is that drug resistance may occur and such resistance is the main reason that makes cancer treatment difficult. One of the drug resistance mechanisms derived from the drug efflux system of cells. ATP-binding cassette subfamily G2 (ABCG2) is a family of multi-drug resistance proteins, which protect cell from exogenous and endogenous toxin through the efflux system [13]. ABCG2 interacts with heme structure and porphyrins and block their accumulation in cells and tissues [14, 15]. Intracellular uptake of photosensitizer is accordingly regulated by ABC transporter superfamily member ABCG2. ABCG2 was regarded as the main cause of resistance to PDT. Studies with ABCG2 knock-out mice had shown the accumulation of porphyrin photosentizer and increase in photosensitivity compared to wild type mice, which means that the ABCG2 protein plays a major role in PDT efficacy [16]. Also, the overexpression of ABCG2 induced resistance to PDT by blocking the accumulation of photosensitizer in cell [13]. In colon cancers, a study reported that the expression of ABCG2 correlated with the shortened patient survival [17].

In this study, we hypothesized that ABCG2 is a target protein in enhancing colon cancer PDT efficacy. To confirm our hypothesis, we tested PDT effect depending on ABCG2 expression level. We also checked the results after the inhibition of ABCG2 with Ko-143. In order to show that ABCG2 is directly related with PDT efficacy, we established ABCG2 overexpressing stable cells in SW480 and tested PDT efficacy in vitro. Furthermore, we checked the anti-tumor effect on xenograft model using cells with either low or high expression of ABCG2 cell in vivo. The results from this study show a possible cause of reduced sensitivity to photosensitizer-PDT in colon cancer cells. Inhibition of ABCG2 expression may be a potential solution to enhance photosensitivity.

## Methods

### Materials

Pheophorbide a (PPa) was obtained from Frontier Scientific Inc. (USA). Ko-143 and thiazolyl blue tetrazolium bromide (MTT) were from Sigma Aldrich. Mouse monoclonal antibodies to ABCG2 and β-actin were from Santa Cruz (USA). Polyclonal rabbit antibody to caspase (cleaved) was purchased from abcam (UK). Polyclonal rabbit antibody to LC3 was purchased from Cell Signaling Technology (USA).

### Cell culture and in vitro photodynamic treatment

SW480, SW480/ABCG2, HT29, HCT116, LoVo, and DLD1 cells were maintained under an atmosphere of 5 % CO2 in RPMI medium (Genedepot, USA) supplemented with 10 % fetal bovine serum and 1 % penicillin-streptomicin (Genedepot, USA). Cells were incubated with or without PPa (0–400 nM) for 16 h at 37 °C in RPMI medium supplemented with 5 % fetal bovine serum and 1 % penicillin-streptomicin. Ko-143 (0-1uM) were pretreated or treated for 1 h at 37 °C. The cells were then photoirradiated using a diod laser emitting red light at 670 nm wavelength (equipment by Kuk-Je A & SL Co., Seoul, Korea). The power density at the illumination area was 800 mW/cm^2^ and total light dose was 4 J/cm^2^. Thereafter, cells were harvested at 4 h, 8 h, and 24 h, respectively.

### Cell viability assay

Cells were cultured in 96-well culture plates (1 × 10^4^cells/well) overnight. Cells applied to PDT were incubated for 4 h with 0.5 mg/ml thiazolyl blue tetrazolium bromide. Converted MTT formazan crystals were solubilized DMSO. The absorbance at 540 nm was measured using microplate reader (Biotek ELX-800 Absorbance Reader, USA).

### Measurement of Singlet oxygen production

Cells were incubated with or without PPa (0–400 nM) for 16 h at 37 °C, washed twice in PBS, and lysed in methanol. Lysed cells were applied to PMT-based singlet oxygen monitoring system (Physical Sciences Inc., USA).

### Quantitative Real-time PCR

Total RNA was isolated with Trizol Reagent (Invitrogen, USA) and reverse-transcribed with a reverse transcription (RT)-PCR kit (Takara) according to manufacturer’s instruction. The level of ABCG2 was measured using a MX-3000P (Stratagene, USA). SYBR green master mix (Takara, Japan) was used to quantify the mRNA expression of ABCG2. The primers used for quantitative RT-PCR were as follows: ABCG2 sense, 5’-TGGCTTAGACTCAAGCACAGC; ABCG2 antisense, 5’-TCGTCCCTGCTTAGACATCC-3; GAPDH sense, 5’-AGCCACATCGCTCAGACAC-3’; GAPDH antisense, 5’-GCCCAATACGACCAAATCC-3’.

### Immunocytochemical assay

Cells were cultured on glass coverslip. Glass coverslips of confluent cells were washed with PBS two times. Cells were fixed for 20 min at room temperature in 4 % paraformalaldehyde/PBS. After rinsing with PBS, cells were incubated with 5%FBS/PBS for 30 min to block non-specific staining. After washing with PBS, cells were incubated with ABCG2 antibody in 5%FBS/PBS overnight (1:200). After washing with PBS, cells were incubated with secondary Alexa-488 antibody in 5%FBS/PBS for an hour. Cells were monitored by fluorescence microscopy (Axiovert 200 MAT, Zeiss, Germany).

### Flow cytometry

Cells were applied to PDT and then incubated with Annexin V-FITC or PI for 15 min at room temperature. Measurement of apoptosis or necrosis was done using FACS Calibur flow cytometer (BD Bioscience, USA).

### Immunoblot analysis

Cells lysed in buffer containing 20 mM HEPES (pH 7.0), 1 % Triton X-100, 150 mM NaCl, 10 % glycerol, 2 mM EGTA, 1 mM EDTA, 1 mM Glycerol 2-phosphate, 1 μg/ml leupeptin, 1 μg/μl aprotinin, 1 mM AEBSF, 50 mM NaF, and 1 mM NA_3_VO_4_. The proteins separated by SDS-PAGE were transferred to a nitrocellulose membrane using electrophoresis tank. After the membrane was incubated with specific antibodies, the signal was enhanced with chemiluminesence reagents (Genedepot) and then measured by LAS-3000 Image Analysis System (Fujifilm, Japan).

### Stable cell establishment in SW480 cells using retrovirus

For preparation of SW480/ABCG2, retrovirus encoding ABCG2 genes were produced by using BglII/XhoI sites. HEK293T cells were transfected with pMSCV-ABCG2, pgag-pol, and pVSV-G, using Lipofectamine2000, and then 48 h later, media including Prx III retroviruses were collected and filtered to remove cell debris. SW480 cells were incubated with ABCG2 retrovirus, and the cells were inoculated with ABCG2 retrovirus. The cells expressing ABCG2 were selected with puromycin.

### Xenograft model and Photodynamic therapy

Four-week-old male BALB/c nude mice were used for in vivo study. The animal experiments were conducted in accordance with the institutional guidelines of the Catholic University of Korea, College of medicine, Seoul, Korea. SW480 and SW480/ABCG2 cells (0.5 × 10^6^) were inoculated subcutaneously in 100 μl of phosphate-buffered saline (PBS). The mice were then divided into a treatment and a control group. The treatment group consisted of two subgroups: the control group and the PDT applied group (670 nm). Animals were injected i.v. with Ko-143(10 mg/kg) followed by i.v. PPa after 1 h.

### Statistical analysis

Data were expressed as means ± standard error of mean (SEM) using Student *t* test.

## Results

### Differences of PDT effect derived from PPa accumulation depending on ABCG2 expression level in colon cancer cell

**Fig. 1:**
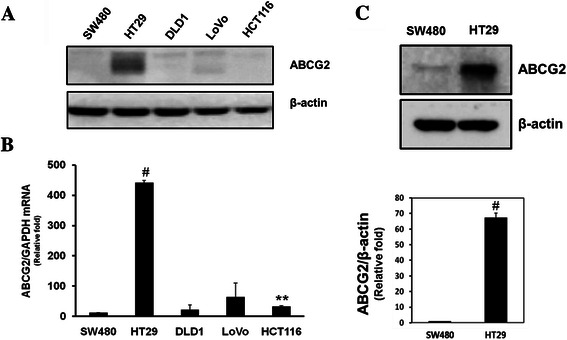
ABCG2 expression level in colon cancer cell lines. **a**, Immunoblotting analysis of whole cell lysates of various colon cancer cell lines. **b**, Total RNA was isolated from colon cancer cell lines, reverse-transcribed, and quantified by quantitative real-time PCR with the specific primer for ABCG2 and GAPDH, respectively **c***,* SW480 and HT-29 cell lysates were subjected to immunoblot analysis with indicated antibodies. Values were expressed as fold change relative to control and were normalized to actin. Data are means ± SEM from three independent experiments (^*^P < 0.05, ^**^P < 0.01, ^#^P < 0.0001)

**Fig. 2:**
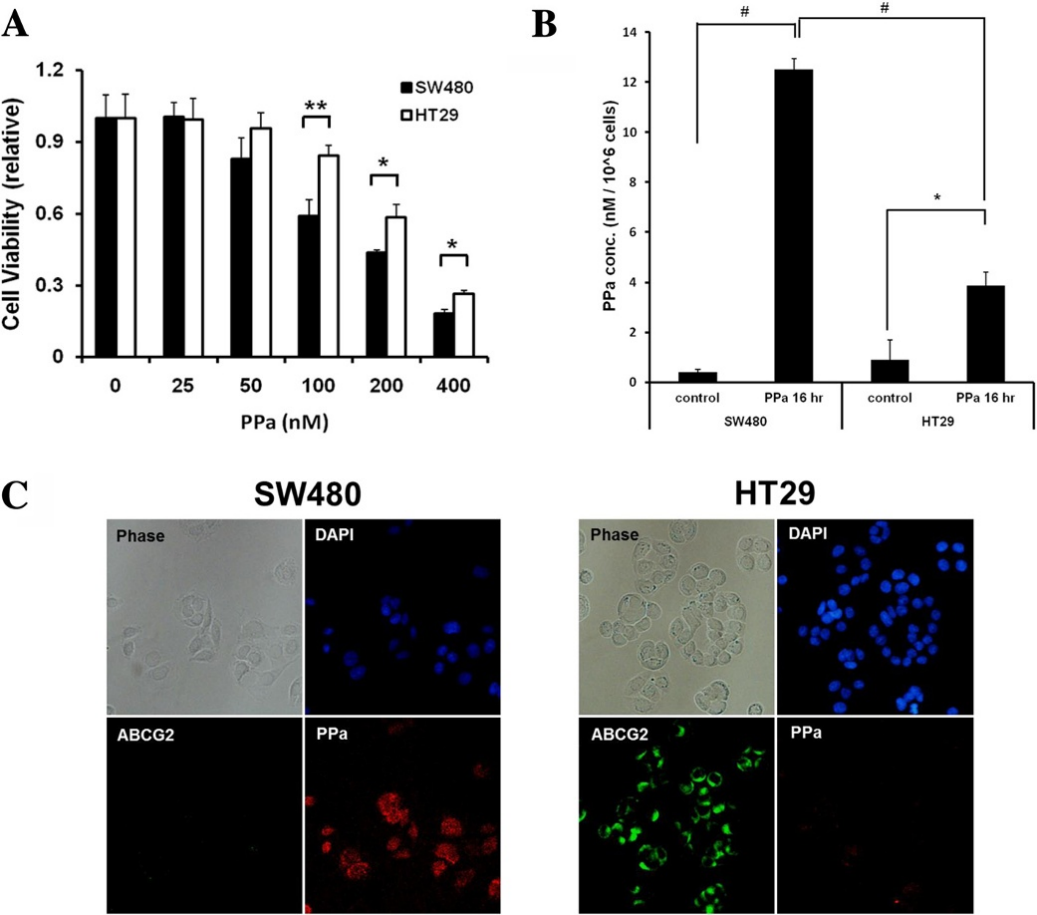
Differences of PDT effect between SW480 and HT29 cells depending on ABCG2 expression level. **a**, SW480 and HT29 cells were irradiated with a PDT laser (4 J/cm^2^) after a 16 h pretreatment of PPa at indicated concentrations. The MTT assay was performed in triplicate 24 h following the irradiation. **b**, Cells were incubated with 100 nM PPa for 16 h and then lysed with methanol, followed by application of PMT-based singlet oxygen monitoring system. **c**, Cells were incubated with 200 nM PPa for 16 h and then subjected to immunocytochemical staining for anti-abcg2 (Green), PPa (Red), and DAPI (blue). Data are means ± SEM from three independent experiments (^*^P < 0.05, ^**^P < 0.01, ^#^P < 0.0001)

The aim of this study was to see whether ABCG2 is a target protein in enhancing colon cancer PDT efficacy. To confirm ABCG2 effect in PDT, we tested ABCG2 expression level in colon cancer cell lines (Fig. 1a, b). In colon cancer cells, HT29 cell showed the highest expression of ABCG2 mRNA and protein. SW480, DLD1, LOVO, and HCT116 cells showed low expression of ABCG2. Among them, SW480 cells showed the lowest ABCG2 mRNA level. SW480 and HT29 cells were selected, which showed the lowest and highest ABCG2 expression level, respectively, among the tested colon cancer cells (Fig. 1c). Cells were incubated with PPa for 16 h and then irradiated with 4 J/cm^2^ red right. There were differences in the cell survival rate and singlet oxygen production between SW480 and HT29. Cell survival rate was measured using MTT assay. After PDT, higher treatment efficacy was obtained in SW480 cells compared to HT29 (Fig. 2a). After treating with 100 nM PPa, there was differences of three times in phototoxicity between SW480 and HT29. Singlet oxygen played a main role in killing cancer cells in PDT. We checked the singlet oxygen production using PMT-based singlet oxygen monitoring system. SW480 cell showed lower production rate of singlet oxygen than HT29 (Fig. 2b). These results indicate that high expression of ABCG2 induced PPa release out of the cell by the efflux function. To further explain the effect of ABCG2 in PDT, we verified localization of ABCG2 and accumulation of PPa using light fluorescence microscopy (Fig. 2c). SW480 cells showed no fluorescence of ABCG2, but strong red fluorescence of PPa. In contrast, HT29 cells showed ABCG2 fluorescence without accumulation of PPa. These results indicate that ABCG2 is related with the resistance to PDT derived from the efflux of photosensitizer in colon cancer.

### Enhanced efficacy of PDT by ABCG2 inhibition

**Fig. 3:**
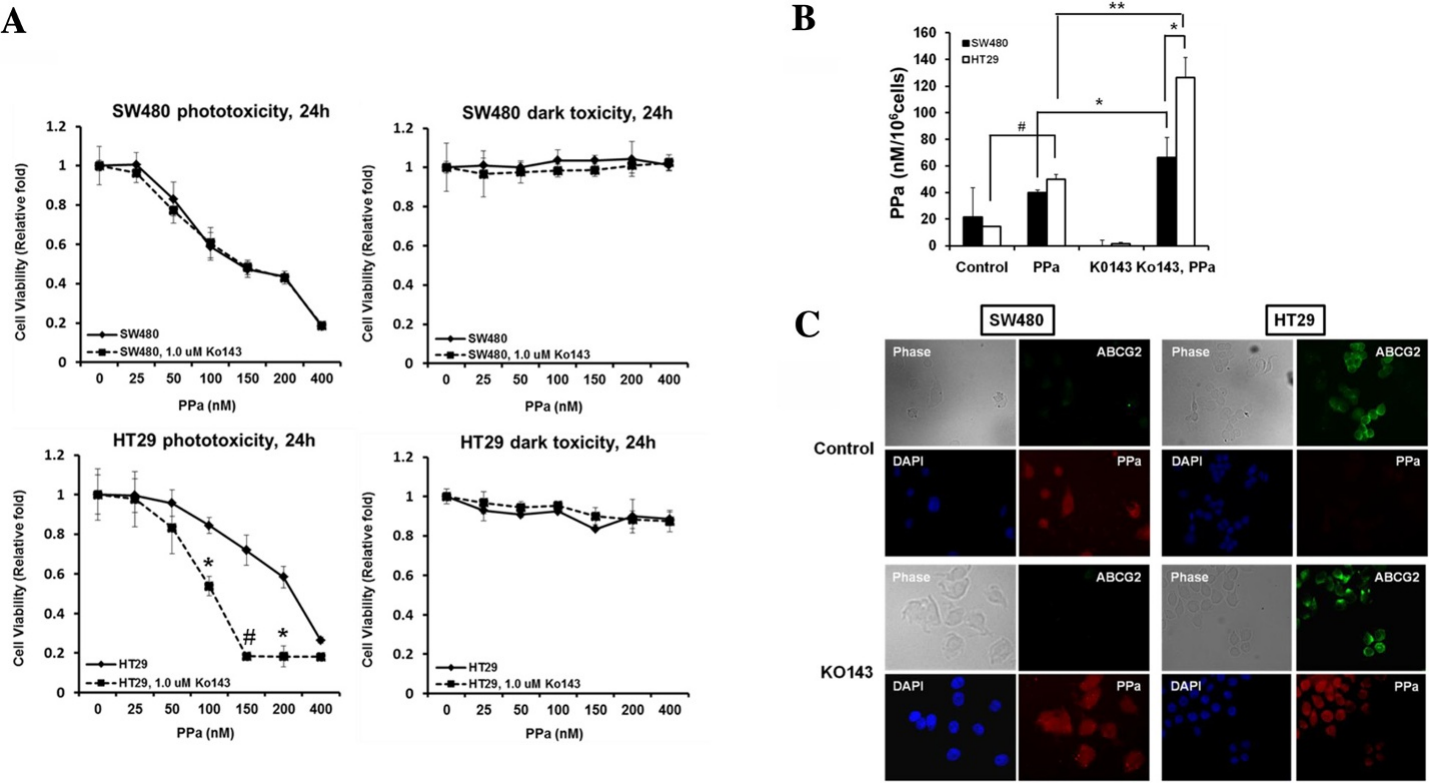
Effect of Ko-143 on PPa treated colon cancer cell in PDT. **a**, Cells were irradiated by 670 nm light (4 J/cm^2^) after 16 h incubation with indicated concentration of PPa in the presence or absence of 1 μM Ko-143. At 24 h after PDT, the MTT assay was performed. **b**, Cells were incubated with 100 nM PPa for 16 h in the presence or absence of 1uM Ko-143 and then lysed with methanol, followed by application of PMT-based singlet oxygen monitoring system. **c**, Cells were incubated with 200 nM PPa for 16 h in the presence or absence of 1uM Ko-143 and then subjected to immunocytochemical staining for anti-abcg2 (Green), PPa (Red), and DAPI (blue). Data are means ± SEM from three independent experiments (^*^P < 0.05, ^**^P < 0.01, ^#^P < 0.0001)

The above findings proved that ABCG2 plays a major role in the resistance of PDT, which could be prevented by using Ko-143, an inhibitor of ABCG2 transporter [18]. To confirm whether the blockage of ABCG2 could increase the effect of PDT in colon cancer, we tested the cell survival rate and singlet oxygen production. Cells were pretreated with 1 μM of Ko-143 for 1 h and then incubated with PPa. There was no change in the SW480 cell survival rate after the inhibition of ABCG2 (Fig. 3a). Contrastingly, HT29 cells showed decreased cell survival rate derived from ABCG2 protection (Fig. 3a). Combined treatment of PPa with Ko-143 enhanced the sensitivity of HT29 cell to PDT. To further investigate the ABCG2 inhibition effect, we measured singlet oxygen production of ABCG2 treated cells and non-treated cells. Singlet oxygen production rate was increased afterABCG2 inhibition in both SW480 and HT29. SW480 cells showed a little increase in the singlet oxygen, with no effect on cell survival rate. On the other hand, HT29 cells treated with Ko-143 showed more singlet oxygen production compared to other cells (Fig. 3b). To clarify that ABCG2 was inversely related with PPa accumulation, we measured fluorescence of PPa in Ko-143treated cells using fluorescence microscope. The level of PPa fluorescence in HT29 cells was remarkably increased by Ko-143 (Fig. 3c). It seems that ABCG2 inhibition increased the sensitivity to PDT by blocking PPa efflux and thereby inducing high level of singlet oxygen.

### Effect of ABCG2 overexpression

**Fig. 4:**
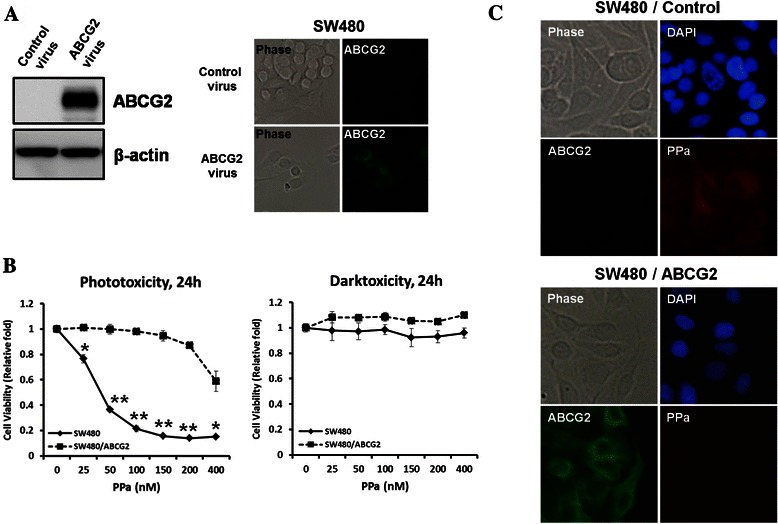
Effect of ABCG2 on the uptake of photosensitizer in SW480 and SW480/ABCG2 cells. **a**, Overexpression of ABCG2 in SW480 was confirmed by immunoblotting and immunocytochemical assay using ABCG2 antibody. **b**, MTT assay was performed in SW480 and SW480/ABCG2 cells. **c***,* SW480 and SW480/ABCG2 cells were incubated with 200 nM PPa for 16 h and then immunocytochemical assay for anti-abcg2 (Green), PPa (Red), and DAPI (blue) was applied. Data are means ± SEM from three independent experiments (^*^P < 0.05, ^**^P < 0.01, ^#^P < 0.0001)

**Fig. 5:**
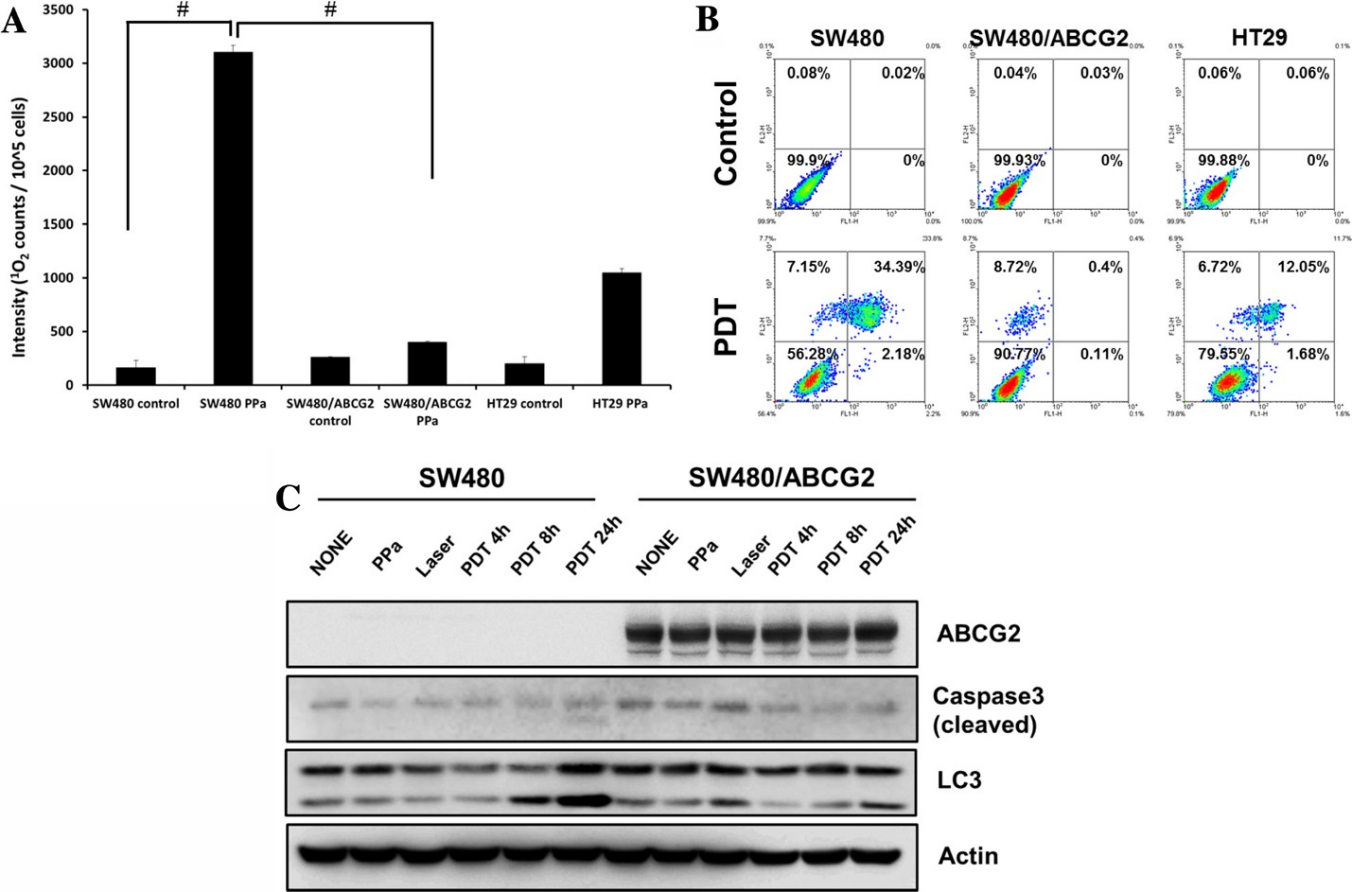
Differences of singlet oxygen production rate & cell death between SW480 and SW480/ABCG2 cells. **a**, SW480, SW480/ABCG2, and HT29 cells were incubated with 100 nM PPa for 16 h and then lysed with methanol, followed by application of PMT-based singlet oxygen monitoring system. **b**, Cells were harvested 4 h after photosensitization and stained with Annexin V/PI for FACS analysis. **c**, Cells were incubated with 100 nM PPa for 16 h followed by irradiation. Cell lysates were subjected to immunoblot with indicated antibodies. Data are means ± SEM from three independent experiments (^*^P < 0.05, ^**^P < 0.01, ^#^P < 0.0001)

As mentioned above, ABCG2 plays a major role in inducing drug resistance and determining the efficacy of PDT. In order to show that ABCG2 is directly related with PDT efficacy, we established SW480 that stably overexpress ABCG2 using retrovirus. The expression level of ABCG2 in SW480 and SW480/ABCG2 was confirmed by western blot analysis and immunocytochemistry with anti-ABCG2 antibody (Fig. 4a). SW/ABCG2 cells with overexpression of ABCG2showed higher level of survival rate compared to SW480 and HT29, which indicates that overexpression of ABCG2 is directly related with the efficacy of PDT and drug resistance (Fig. 4b). SW480/ABCG2 cells showed low fluorescence of PPa, whereas SW480 cell showed strong signal of PPa (Fig. 4c). Also, singlet oxygen production rate was decreased in SW480/ABCG2 cells (Fig. 5a). To investigate the effect of PDT on cell viability, we checked for apoptosis or necrosis using flow cytometry analysis with Annexin V and PI. After irradiation, apoptosis and necrosis developed considerably in SW480. SW480 cells with overexpression of ABCG2 showed little necrosis while HT29 cells showed some areas of apoptosis and necrosis (Fig. 5b). In order to verify the effect of ABCG2 on PDT efficacy, we checked for the cleaved caspase-3 and LC3II level. Cleaved caspase-3 is known as a marker of apoptosis and LC3II suggests autophagic cell death [19, 20]. Cleaved caspase-3 level in SW480 was further increased at 24 h after PDT than SW480/ABCG2 (Fig. 5c) and SW480/ABCG2 cells showed low level of LC3II than SW480 at post-24 h PDT (Fig. 3c). The above data indicate that ABCG2 expression level in colon cancer cells is inversely related with PDT efficacy, and that ABCG2 inhibition could therefore increase cancer cell death.

### *In vivo* antitumor effects of PDT

**Fig. 6:**
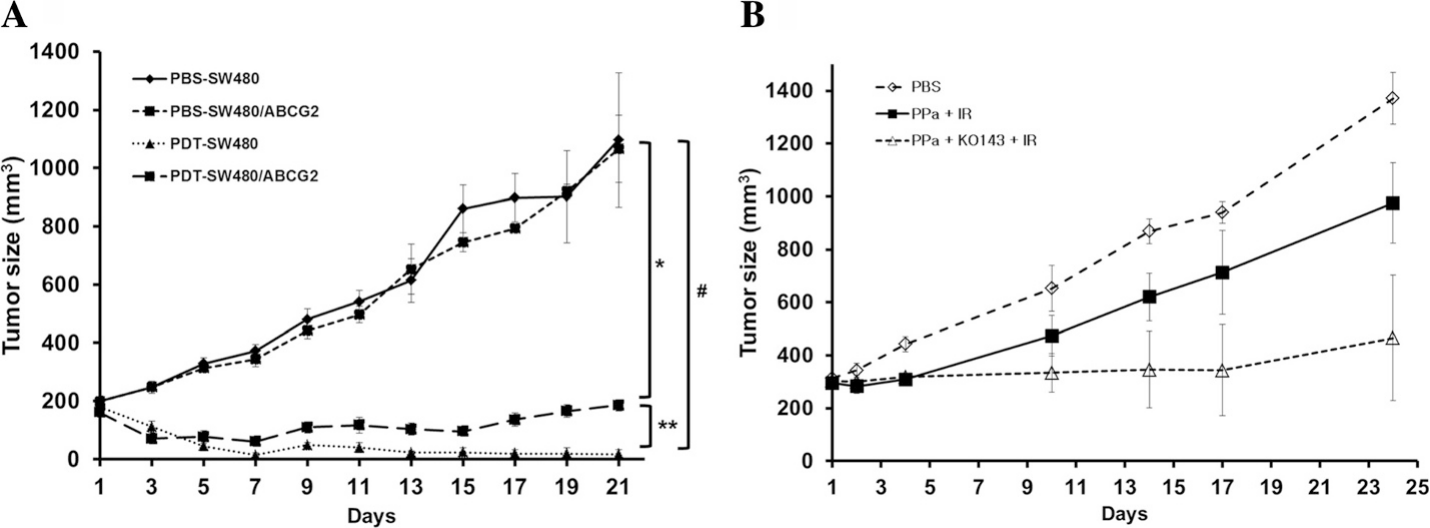
Antitumor effects of PDT in *in vivo* experiments. **a**, SW480 and SW480/ABCG2 cells were collected and subcutaneously injected into BALB/c nude mice. The sizes of tumors were measured using a caliper, tumor volume was calculated using the formula: 0.523 × length × width^2^ (mm^3^). **b**, HT-29 cells xenografted in nude mouse were treated with PBS alone, PPa + irradiation with or without Ko-143 pretreatment. Combined treatment of PPa with Ko-143 enhanced the sensitivity of HT29 cell to PDT. Data are means ± SEM from *n* = 4 (^*^P < 0.05, ^**^P < 0.01, ^#^P < 0.0001)

To test for the effects of ABCG2 expression in vivo, PDT was applied to xenograft tumor models established by subcutaneous injection of colon cancer cells (SW480, SW480/ABCG2). When tumor size reached150-200 mm^3^, PPa was administered to the tumors at the dose of 1.25 mg/kg followed by irradiation with a 670 nm diode light (150 J/cm^2^) 6 h later. There was no change in size of the tumor between SW480 and SW480/ABCG2 injected mice immediately after PDT. However, at 7 days after the injection, the tumor volume increased by a greater extent in mice injected with SW480/ABCG2 cells compared to mice injected with SW480 cells (Fig. 6a). To confirm this result, HT-29 cells xenografted in nude mouse were treated with PBS alone, PPa + irradiation with or without Ko-143 pretreatment (Fig. 6b). As shown in cell experiments, combined treatment of PPa with Ko-143 enhanced the sensitivity of HT29 cell to PDT. These results show that ABCG2 expression influences the outcome of PDT in vivo.

## Discussion

In this study, we demonstrated that ABCG2 plays a main role in drug resistance related to PDT in colon cancer cell. Our data showed that ABCG2 overexpression strongly induced photosensitizer efflux which means that ABCG2 inhibition may be the solution of drug resistance in colon cancer. Studies indicated that ABCG2 contributes to protection of cellular accumulation of porphyrin and toxic heme components [14]. ABCG2 was first discovered in a breast cancer cell line, which is also called breast cancer resistance protein (BCRP/ABCG2) [21]. It has been reported that ABCG2 overexpression using temporarily ABCG2-transfected HEK293 cells showed decreased cellular accumulation of PPIX, whereas other ABC-transporter overexpression (ABCB1 and ABCC1) did not effect on PPIX accumulation, which suggested that ABCG2 could be the main target of drug resistance [12]. In human colon cancer cell, ABCG2 is known as main cause of resistance to drug such as SN-38 which is used for chemotherapy in colon cancer [22]. ABCG2 is important in developing drug resistance in PDT. However, the association ABCG2 and PDT efficacy in colon cancer cell remain unclear. Our studies demonstrate that PDT efficacy is regulated by ABCG2 expression level in colon cancer. First of all, we checked the ABCG2 expression level in mRNA and protein. We found that high expression level of ABCG2 cells showed low efficacy of PDT which was resulted from low accumulation PPa due to ABCG2 induced efflux. Fluorescence microscopy experiments also showed low level of PPa in ABCG2 overexpressed cell. Also, ABCG2 inhibitor, Ko-143, treated cell showed increased PPa accumulation, which induced high PDT efficacy. Consistently, ABCG2 knock-down in HT-29 using siRNA showed increased PPa (data not shown). PDT efficacy was correlated with ABCG2 expression level. SW480/ABCG2 expressing high level of ABCG2 showed no apoptosis or necrosis after PDT, whereas, SW480 cells were prone to apoptosis or necrosis. These phenomena were also confirmed by higher level of cleaved caspase-3 and LC3II in SW480 cells. SW480 treated with PDT showed increased cleaved caspase-3 and LC3II compared to SW480/ABCG2, which means that apoptotic and autophagic cell death was induced in SW480 cells. Tumor cell death in PDT was induced by the release of singlet oxygen produced by photo-excitation of photosensitizer by visible light [23]. We found that singlet oxygen production was correlated with PPa accumulation in cells. These data suggested that PDT efficacy was dependent on PPa accumulation and PPa induced singlet oxygen production. According to recent report, ABCG2, drug transporter, regulates the outcome of hypericin-mediated photodynamic therapy in HT-29 [24].

ABCG2 has been already found to have the main role in porphyrin accumulation in mice. To show ABCG2 directly influenced PDT efficacy, we established SW480 stably overexpressing ABCG2 and also tested PDT efficacy using in vitro system, and then we checked the anti-tumor effect on xenograft model using cells differently expressing ABCG2 in vivo studies. The tumor size of SW480 cells was significantly smaller in the group treated with PPa-PDT compared to the SW480/ABCG2 injected group. These results indicate that antitumor effects are dependent on ABCG2 expression level.

Many researchers reported that ABCG2 inhibition had decreased PDT efficacy through other protein regulation [25–27]. However, we directly showed ABCG2 effect in colon cancer cell using ABCG2 overexpressed cell line both *in vitro* and *in vivo,* providing direct evidences of enhanced PDT effect when inhibiting the ABCG2. In this aspect, our research suggests a very important role of ABCG2 in drug resistance and PDT efficacy in colon cancer cell, and provides reasonable and sufficient evidence to develop ABCG2 inhibitor for protecting drug resistance.

## Conclusion

In summary, this study shows a possible cause of reduced sensitivity to photosensitizer-PDT in colon cancer cells. Our study provides a potential solution to enhance photosensitivity by inhibition of ABCG2 expression.
